# Comparative efficacy of certoparin, enoxaparin, and combined thromboprophylaxis on thromboembolic events after glioblastoma resection: a prospective observational study

**DOI:** 10.1038/s41598-025-07899-2

**Published:** 2025-07-01

**Authors:** Eva Wardenbach, Dino Podlesek, Boshr Alhasan, Ahmed Abouelhamd, Ilker Y. Eyüpoglu, Tareq A. Juratli, Witold H. Polanski

**Affiliations:** 1 Department for Neurosurgery, Faculty of Medicine and University Hospital Carl Gustav Carus, TU Dresden University of Technology, Fetscherstraße 74, Dresden, 01307 Germany; 2 Clinic for Orthopedics and Spinal Surgery, Fichtelgebirge Hospital, Marktredwitz, Bavaria Germany

**Keywords:** Thromboembolic events (TE), Glioblastoma, Anticoagulant treatments, Postoperative complications, Risk factors, Outcomes research, Cancer, Surgical oncology, Medical research, Oncology, Risk factors, Cancer

## Abstract

Thromboembolic events (TE) are serious complications following glioblastoma (GBM) resection. This retrospective study analyzed 695 GBM patients (2017–2022, University Hospital Dresden) to assess the impact of different anticoagulant regimens—certoparin, enoxaparin, and enoxaparin with intermittent pneumatic stockings (IPS)—along with patient comorbidities, on TE incidence. Overall, 28 patients (4%) developed TE. The highest combined incidence of deep vein thrombosis (DVT) and pulmonary embolism (PE) occurred in the enoxaparin group (8.6%), followed by certoparin (6.9%) and enoxaparin + IPS (2.6%) (*p* = .003). Increased PE risk was associated with longer surgery duration (median 249 vs. 190 min; *p* = .002), greater intraoperative blood loss (300 vs. 150 mL; *p* = .002), and older age (> 65 years, *p* = .043). Comorbidities such as diabetes (*p* = .005) and coronary heart disease (*p* = .037) were also linked to elevated TE risk. Multivariate analysis identified enoxaparin alone as an independent risk factor (HR 0.312; CI 0.116–0.842; *p* = .022). Patients with PE or DVT had surgeries that were on average 45 min longer and involved higher blood loss. GBM patients treated with enoxaparin alone have a significantly higher risk for TE compared to treatment with certoparin or the combination of enoxaparin with IPS. Additionally, the duration of surgery, patient age and comorbidities significantly influence the risk of postoperative TE.

## Introduction

Glioblastoma WHO grade 4 (GBM) is the most prevalent and aggressive primary malignant brain tumor in adults, characterized by rapid progression and a poor prognosis^1^. Despite significant advances in surgical techniques, radiation and chemotherapy, the median survival remains limited to approximately 12 to 15 months after diagnosis^2–4^. In Germany, the incidence of GBM is approximately 6 per 100,000 individuals, accounting for around 5,000 new cases per annually^5^. The aggressive nature of the disease and the invasiveness of surgical interventions contribute to high morbidity and mortality rates^6,7^.

Postoperative thromboembolic events (TE) such as deep vein thrombosis (DVT) and pulmonary embolism (PE) are serious complications frequently observed in neurosurgical patients, particularly those undergoing GBM resection^8–11^. The risk of TE in these patients is notably elevated due to a combination of factors, including prolonged immobility, surgical trauma and the prothrombotic state induced by cancer itself^12,13^. Existing literature reports incidence rates of DVT as high as 60% and PE rates up to 10% in GBM patients without thrombosis prophylaxis, with mortality from PE reaching 50%^3,12,14–16^. These events significantly impact patient outcome and quality of life, adding to the complexity of postoperative care^17^. The current recommendations of thromboprophylaxis in neurosurgical patients are inconsistent and vary across institutions^5,18–21^. While mechanical prophylaxis, such as intermittent pneumatic stockings (IPS), is widely endorsed, pharmacological prophylaxis using low-molecular-weight heparins (LMWH) remains controversial and imprecise due to concerns about intracranial hemorrhage^11,18^. The S3 guideline for thromboprophylaxis offers only limited recommendations for.

neurosurgical patients, reflecting the lack of robust data specific to this group^21^. Moreover, no anticoagulants are officially approved for the use in neurosurgical patients by most manufacturers due to the perceived high risk of bleeding associated with central nervous surgery^22,23^. This open recommendation is based on a survey of 34 neurosurgical centers, which revealed that approximately 90% of clinics provide their patients with LMWH postoperatively^24^. There is a strong recommendation for mechanical prophylaxis, for example, the use of IPS. The benefit of this approach was clearly demonstrated in several prospective, randomized studies and justified in a metanalysis of 30 publications of 7779 patients who underwent neurosurgical surgery^18^. Given these challenges, there is an urgent need to evaluate the efficacy and safety of various thromboprophylaxis strategies in patients undergoing GBM surgery^11,14^. Recently, a systematic review by Bianconi et al. (2023)^25^ highlighted the ongoing debate surrounding anticoagulation management in high-grade glioma patients, emphasizing the challenging balance between preventing TE and avoiding intracranial hemorrhage.

This study aimed to investigate the effectiveness of different thromboprophylaxis strategies, specifically comparing pharmacological and mechanical interventions. Patients with comorbidities or a history of TE were deliberately not excluded, allowing for a comprehensive and realistic assessment of potential risk factors. To address existing knowledge gaps, the analysis specifically focused on certoparin, enoxaparin, and the combination of enoxaparin with IPS, assessing their respective impacts on the incidence of postoperative symptomatic TE. Furthermore, patient-specific and procedure-specific risk factors were examined to improve risk stratification in this high-risk population. The overarching goal was to develop evidence-based recommendations that can effectively guide clinical practice, enhancing patient outcomes by reducing symptomatic TE while carefully managing the risk of hemorrhagic complications. Given the understudied nature of GBM patients, who are notably vulnerable both to symptomatic TE and bleeding complications^26^. By evaluating the outcome associated with these strategies, we hope to inform future guidelines and help optimize postoperative care for patients undergoing neurosurgical procedures.

## Methods

### Study design and population

This prospective observational study consists of 695 patients with newly diagnosed glioblastoma, who underwent surgical treatment between February 2017 and June 2022.

Eligibility required patients to be at least 18 years old with histologically confirmed GBM according to the previous WHO classification. Surgical resection was performed in all cases unless contraindications such as tumor size, location or intraoperative complications like excessive bleeding were present. Patients who underwent biopsy only without surgical resection were excluded from this analysis. The cohort size was determined by the complete data available for all GBM surgeries conducted during the study period. This cohort size was sufficient for identifying statistically significant associations between thromboprophylaxis strategies and TE, as well as for conducting subgroup analyses. The cohort size allowed for robust statistical analysis, while the inclusion criteria ensured a representative sample of the target population. Detailed demographic characteristics are summarized in Table 1.

**Table 1:** Baseline characteristics of glioblastoma patients stratified by thromboprophylaxis group (Group 1: certoparin; group 2: enoxaparin; group 3: enoxaparin + IPS).

Characteristic	Value
Age (years), median	64 (20–94)
Female sex, n (%)	264 (38)
Comorbidities, n (%)	
Epilepsy	301 (43.3)
Arterial hypertension	415 (59.7)
Diabetes mellitus 1/2	106 (15.3)
Chronic heart disease	46 (6.6)
Atrial fibrillation	53 (7.6)
Prior thromboembolic event	48 (6.9)
Clinical parameters, median	
Duration of surgery (min)	191
Blood loss (ml)	150
Thromboembolic complications, n (%)	
Deep vein thrombosis	21 (3)
Pulmonary embolism	20 (2.9)
Deep vein thrombosis/pulmonary embolism	28 (4)

Continuous variables are presented as mean ± SD or median [IQR] depending on distribution; categorical variables as number (%).

Group differences were analyzed using chi-square or Kruskal–Wallis tests as appropriate.

*DVT* deep vein thrombosis, *PE* pulmonary embolism, *CHD* coronary heart disease, *IPS* intermittent pneumatic stockings, *SD* standard deviation, *IQR* interquartile range.

p values < 0.05 are considered statistically significant.

### Thromboprophylaxis interventions

This study assessed three distinct thromboprophylaxis regimens:

1. Certoparin group (*n* = 304): Patients received certoparin, a LMWH as pharmacological prophylaxis.
2. Enoxaparin group (*n* = 163): Patients received enoxaparin for anticoagulation.
3. Enoxaparin combined with IPS (*n* = 228): Patients received enoxaparin in combination with mechanical prophylaxis using IPS.

Patients were assigned to thromboprophylaxis groups based on the institutional standards in effect at the time of their surgery. During the observation period, hospital-wide thromboprophylaxis protocols were revised, leading to a transition from certoparin to enoxaparin, and the introduction of intermittent pneumatic stockings (IPS) as an adjunct measure. These protocol changes were implemented uniformly across the department and are reflected in the group allocations of this study. Group assignment was therefore not based on individual clinical decisions but followed the applicable standard operating procedures during each phase of the study period. All patients were followed for three months postoperatively, and data on the incidence of symptomatic DVT and PE were collected. No routine screening for asymptomatic TE was performed; only symptomatic cases were detected according to existing standard operation procedures using duplex ultrasound or computer tomography.

### Data collection and risk factors

The investigators had full access to the electronic medical records (EMRs) of all GBM patients treated at the University Hospital Dresden during the study period. These records provided comprehensive data on demographics, clinical characteristics, surgical parameters, and postoperative outcomes, authorized through institutional approval and ethics committee supervision (approval number EK63022018). The centralized EMR system ensured reliable and complete datasets for analysis, without missing data on key variables. Efforts to minimize bias included systematic data collection by trained staff, excluding patients with incomplete follow-up, and confirming symptomatic TE using established diagnostic methods like duplex ultrasound and CT, to ensure consistency and accuracy. The study included all eligible patients, accounting for confounders such as comorbidities and procedure-specific parameters using multivariate logistic regression. Patient-specific risk factors analyzed included age, gender, comorbidities (e.g., epilepsy, diabetes, chronic heart disease (CHD), atrial fibrillation, hypertension), medication, and prior TE history. Procedure-specific factors included surgery duration, intraoperative blood loss, postoperative thromboprophylaxis, and the use of IPS versus non-pneumatic stockings.

### Outcome measures

The primary outcome measure was the occurrence of symptomatic DVT, PE or both within three months post-surgery. Symptomatic DVT was diagnosed clinically by pain, swelling, tenderness, warmth, or erythema of an extremity, confirmed by duplex ultrasound. Symptomatic PE was identified clinically by dyspnea, chest pain, tachycardia, hypoxia, or hemoptysis, and subsequently confirmed by computed tomography pulmonary angiography.

### Statistical analysis

The statistical analyses were conducted using the IBM SPSS Statistics software, version 29.0. Descriptive statistics were used to summarize patient characteristics and clinical outcomes. Quantitative variables, such as age, surgery duration, and intraoperative blood loss, were initially analyzed as continuous variables and expressed as medians with ranges. Subsequently, these variables were categorized using clinically relevant thresholds from the literature (e.g., surgery duration > 200 min, blood loss > 200 mL) to facilitate subgroup analyses and improve clinical interpretability. Categorical variables were summarized as absolute frequencies and percentages. The relationships between these variables and the risk of thromboembolic events (TE) were explored using descriptive statistics (e.g., medians and interquartile ranges) and hypothesis tests. Pearson’s chi-square test was applied to categorical variables, while the Mann-Whitney U-test was used to compare continuous variables. A p-value < 0.05 was considered statistically significant in all analyses. To identify independent predictors of TE, a multivariate binary logistic regression model was developed. Variables with significant associations in the univariate analysis were included in the regression and refined through backward elimination, progressively removing non-significant factors. Specifically, variables with a p-value < 0.1 in univariate analysis were included in the initial regression model. A stepwise backward elimination procedure was used, removing variables with *p* > .05 to identify independent predictors of symptomatic thromboembolic events. The final model provided adjusted odds ratios (ORs) with 95% confidence intervals (CIs) to quantify the strength of associations between risk factors and TE.

The model’s explanatory power was evaluated using the Nagelkerke R² statistic and the omnibus test of model coefficients, with a p-value < 0.05 indicating satisfactory model performance.

This analytical approach ensured robust control for potential confounders and allowed for clinically relevant insights into the relationships between patient characteristics, surgical factors, and the risk of symptomatic TE.

## Results

### Study population

A total of 695 patients were included in the analysis, comprising 264 women (38%) and 431 men (62%). The median age of the cohort was 64 years (range: 20–95 years). Detailed demographic and clinical characteristics are shown in Tables 1 and 2.

**Table 2:** Incidence of postoperative symptomatic thromboembolic events (TE) within 3 months, stratified by thromboprophylaxis group.

Characteristic	TE positive	TE negative	*p*-value
Median age, year (IQR)	68	63	0.041
Sex, n (%)			
Female	6 (2.3)	257 (97.7)	0.075
Male	22 (5.1)	409 (94.4)	
Comorbidities, n (%)			
Hypertonus (414)	22 (5.3)	392 (94.7)	0.048
Chronic heart disease (46)	4 (8.7)	42 (91.3)	0.107
Epilepsy (301)	10 (3.3)	291 (96.7)	0.442
Diabetes (106)	10 (9.4)	96 (90.6)	0.005
Atrial fibrillation (52)	3 (5.8)	49 (94.2)	0.459
Prior TE (48)	4 (8.3)	44 (91.7)	0.121
Prophylaxis group, n (%)			
Certoparin	8 (2.6)	296 (97.4)	0.003
Enoxaparin	14 (8.6)	149 (91.4)	
Enoxaparin + IPS	6 (2.6)	221 (97.4)	
Blood loss			
Median blood loss (mL)	200	150	0.012
Blood loss > 200 mL, n (%)	13 (6.4)	190 (93.6)	0.059
Duration of surgery			
Median duration (min)	234	189	0.011
Duration > 200 min, n (%)	20 (6.4)	291 (93.6)	0.006

Events are shown separately for DVT, PE, and combined TE.

*DVT* deep vein thrombosis, *PE* pulmonary embolism, *TE* thromboembolic events, *IPS* intermittent pneumatic stockings.

Group 3 (enoxaparin + IPS) showed the lowest combined TE rate.

p values refer to group comparisons using chi-square test.

### Frequency of symptomatic TE (DVT/PE)

In the cohort, 28 patients (4%) experienced symptomatic TE, which included DVT and PE. Specifically, 3% of patients (*n* = 21) developed symptomatic DVT, 2,9% developed symptomatic PE (*n* = 20) and 4% experienced both symptomatic DVT and PE (*n* = 13) within the three-month postoperative period.

### Thromboprophylaxis group as risk of TE

**Fig. 1:**
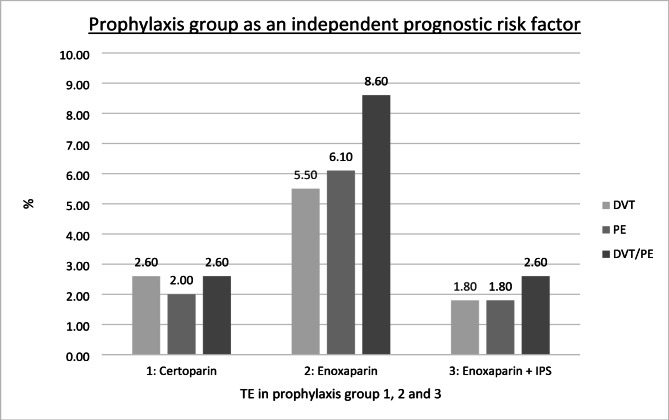
Incidence of symptomatic thromboembolic events (TE) by prophylaxis group. The bar chart shows the proportion of patients developing symptomatic deep vein thrombosis (DVT), pulmonary embolism (PE), or both (DVT + PE) within 3 months postoperatively, stratified by thromboprophylaxis regimen: Group 1 (certoparin), Group 2 (enoxaparin), and Group 3 (enoxaparin + intermittent pneumatic stockings [IPS]). Group 2 exhibited the highest combined incidence of DVT and PE (8.6%) compared to Group 1 (6.9%) and Group 3 (2.6%) (*p* = .003, chi-square test). Multivariate logistic regression confirmed prophylaxis group as an independent predictor for TE (*p* = .022; OR 3.2; 95% CI: 1.2–8.6), with Group 3 serving as the reference. *TE* thromboembolic event, *DVT* deep vein thrombosis, *PE* pulmonary embolism, *IPS* intermittent pneumatic stockings.

The frequency of thromboembolic complications varied significantly across the three thromboprophylaxis groups (Fig. 1). Patients in the enoxaparin group (group 2) exhibited a higher incidence of both symptomatic DVT and PE compared to the certoparin group (group 1), and the enoxaparin combined with IPS Group (group 3). The combined incidence of symptomatic DVT and PE was highest in group 2 (enoxaparin only) with 8.6%, followed by group 1 (certoparin) with 6.9%, and group 3 (enoxaparin + IPS) with 2.6% (*p* = .003). The incidence of symptomatic DVT alone was 5.5% in group 2, 2.6% in group 1, and 1.8% in group 3 (*p* = .088).

### Patient-specific risk factors

Several patient-specific risk factors were identified as significantly associated with the incidence of symptomatic TE. Older patients (aged over 65 years) were found to have higher prevalence of symptomatic PE (4.3%) compared to younger patients (1.6%; *p* = .043). Additionally, patients with a median age of 68 years were more likely to experience symptomatic TE compared to younger patients (*p* = .041).

While GBM was more common in men (p = < 0.001), male sex was not found to be a significant risk factor for symptomatic TE. No statistically significant differences were observed in the incidence of symptomatic DVT (*p* = .25), PE (*p* = .106) or combined symptomatic DVT/PE (*p* = .075) between male and female patients. CHD was associated with higher incidence of symptomatic PE (8.7% in CHD patients vs. 2.5% in non-CHD patients; *p* = .037). Additionally, patients with diabetes mellitus had a significantly increased risk of symptomatic PE (*p* = .006) and combined symptomatic DVT/PE (*p* = .005). The incidence of symptomatic PE in diabetes patients was 7.5%, and 9.4% developed both symptomatic DVT and PE, compared to 2.0% and 3.1% respectively, in non-diabetic patients. Conversely, hypertension has no significant effect on the occurrence of symptomatic DVT (1.8% with no TE vs. 3.9% with DVT, *p* = .17) or symptomatic PE (1.8% with no TE vs. 3.6%, *p* = .17 with PE). However, there is a significant effect on the occurrence of both symptomatic DVT and PE combined (2.1% with no TE vs. 5.3%, *p* = .048 with DVT/PE). Epilepsy was also not associated with a higher risk of any symptomatic TE: DVT: *p* = .662, PE: *p* = .822, DVT/PE: *p* = .442.

A diagnosis of atrial fibrillation was recorded in 7.6% of the cohort. There was no evidence that the presence of atrial fibrillation increased the risk of symptomatic TE, as demonstrated by the following p-values: DVT (*p* = .66), PE (*p* = .65), and DVT/PE (*p* = .459). Similarly, a history of TE did not affect the subsequent incidence of symptomatic TE following surgical resection of GBM: DVT (*p* = .17), PE (*p* = .154), and DVT/PE (*p* = .121).

### Procedure specific risk factors

Procedure-specific factors also played a significant role in the development of symptomatic TE. Longer surgeries were associated with a higher risk of symptomatic PE. Patients who developed symptomatic PE had a median surgical time of 249 min compared to 190 min for the overall cohort (*p* = .002). Additionally, surgeries in patients with combined symptomatic DVT/PE were on average 43 min longer than those without symptomatic thromboembolic complications (*p* = .011).

Intraoperative blood loss was another significant risk factor. Patients who developed symptomatic PE lost a median of 300mL of blood compared to 150mL in patients without symptomatic PE (*p* = .002). Similarly, patients with combined symptomatic DVT/PE experienced greater blood loss (median 200mL; *p* = .012).

The majority of patients (76.8%) were positioned supine during surgery, followed by, prone (14.9%), lateral (8.1%) and Trendelenburg position (0.1%). However, the small sample sizes of the individual subgroups limited meaningful statistical evaluation of these data.

### Independent predictors for symptomatic TE

Multivariate binary logistic regression analysis was performed to identify independent predictors of symptomatic TE (Table 3). Compared to the reference group (enoxaparin + IPS), patients in the enoxaparin-only group had a significantly higher risk of TE (OR 3.2; 95% CI 1.2–8.6; *p* = .022). In addition, diabetes mellitus was identified as an independent predictor for PE (OR 3.6; 95% CI 1.4–9.2; *p* = .007) and combined DVT/PE (OR 3.0; 95% CI 1.3–6.8; *p* = .009). Among the procedure-specific factors, surgery duration > 200 min independently predicted DVT (OR 2.5; 95% CI 1.0–6.4; *p* = .047), PE (OR 2.9; 95% CI 1.0–8.4; *p* = .045), and combined DVT/PE (OR 2.6; 95% CI 1.0–6.5; *p* = .039). Blood loss > 200 mL was also associated with increased PE risk (OR 2.9; 95% CI 1.0–8.4; *p* = .045).

**Table 3:** Multivariate logistic regression analysis of independent predictors for symptomatic thromboembolic events (TE) within 3 months.

	DVT	Sig (*p*)	HR	PE	Sig (*p*)	HR	DVT/PE	Sig (*p*)	HR
Patient specific									
DM 1/2				x	0.007	3.6	x	0.009	3.0
Procedure specific									
Surgery duration	x	0.047	2.5	x	0.045	2.9	x	0.04	2.6
Blood loss				x	0.03	0.35			
Prophylaxis specific									
Enoxaparin							x	0.022	0.3

The reference group for thromboprophylaxis was Group 3 (enoxaparin + IPS). Hazard ratios (HR), 95% confidence intervals (CI), and *p* values are reported.

*DVT* deep vein thrombosis, *PE* pulmonary embolism, *CHD* coronary heart disease, *IPS* intermittent pneumatic stockings.

Variables included were those with *p* < .1 in univariate analysis. *p* values < 0.05 are bolded and considered statistically significant.

## Discussion

### Summary of main findings

The relationship between GBM and thrombosis has become increasingly well-understood through ongoing research. This study emphasizes the need to differentiate between perioperative prophylaxis strategies for GBM patients. In this prospective observational study including 695 glioblastoma patients, we found that the incidence of symptomatic TE within 3 months postoperatively was 4%. The highest incidence occurred in patients who received enoxaparin monotherapy (8.6%), compared to certoparin (6.9%) and enoxaparin plus intermittent pneumatic stockings (IPS; 2.6%). In multivariate analysis, enoxaparin monotherapy, diabetes mellitus, prolonged surgery duration (> 200 min), and intraoperative blood loss (> 200 mL) emerged as independent predictors of TE. No significant association was found for sex, epilepsy, atrial fibrillation, or prior TE history.

### Interpretation of results and comparison with previous literature

Similar to other studies, we found that combining LMWH with IPS yielded the best patient outcome^27^. As there are currently no specific guidelines on the preferred LMWH, and given the efficacy highlighted in our study, further research is needed to fully understand the varying impact of different agents.

While general guidelines exist for postoperative thromboprophylaxis in cancer patients, the selection of a specific LMWH remains a subject of ongoing debate^5,19,20^. Increasing evidence in neurosurgery supports the use of LMWHs, which do not significantly increase the risk of postoperative intracranial hemorrhage when doses are controlled (< 4000I U/day) and administrated the day after surgery^18,28^. To date, no clear recommendations exist regarding the most appropriate LMWH, such as certoparin, enoxaparin, dalteparin or nadroparin, and whether their effectiveness in reducing symptomatic TE varies. Given that LMWHs are derived from different manufacturing processes, resulting in varied molecular compositions, it is unsurprising that they may differ in function. LMWHs share a mechanism of action that involves antithrombin-mediated inhibition of factor Xa, along with several pleiotropic effects, but no two LMWHS are exactly alike^29–31^. For example, enoxaparin products like Clexane (est. 1994) and Inhixa (est. 2017), though both classified as LMWHs, differ in their properties due to their unique production processes. While marketed as biosimilars, these products are not identical. Inhixa, introduced in 2017 under revised European Medicines Agency (EMA) approval processes^32^, was approved through a simplified regulatory process as part of EU harmonization efforts, despite being a complex mixture of biogenic origin. The potential clinical value of a biosimilar can be assessed using the System of Objectified Judgement Analysis (SOJA). In this case, the total score would currently fall significantly below 1000 points, the target value, due to the lack of clinical data^33^. This raises concerns about clinical efficacy and bioequivalence, even between Inhixa and Clexane, both of which are enoxaparin. Due to the varying chemical modifications introduced by each manufacturing process, LMWHs from different manufacturers are not identical chemical entities^29,34,35^. Imberti et al. recommended in their review from 2017 to not follow the changed EMA approval of enoxaparin biosimilars, due to serious concerns regarding efficacy and safety^34^. Studies have shown that Inhixa has only 85% of the tissue factor pathway inhibitor (TFPI) activity of Clexane, suggesting potential differences in therapeutic efficacy^22,30^. On the other hand, in 2020, Fantoni et al. conducted a retrospective observational report on the safety and efficacy of biosimilar enoxaparin in 189 medical and 192 general surgical patients. All surgical patients underwent major abdominal surgery, again highlighting the lack of representation of neurosurgical patients, who were excluded from the studies.

Fantoni et al. found that the incidence of bleeding and VTE was 0.5%, which is comparable to the estimated rates in their literature analysis^36^. Further research is needed to fully understand these differences and their clinical implications, and to re-evaluate and discuss the changes in the EMA approval process. We have not yet compared our subgroups based on Inhixa or Clexane, but this will be an interesting target for the future. Our study found that certoparin was more effective than enoxaparin, raising questions about possible pharmacokinetic differences despite their apparently similar properties such as half-life and bioavailability. Further research is needed to determine whether these differences impact patient outcomes, and ongoing studies should explore the bioequivalence and pharmacokinetics of biosimilars like Inhixa and Clexane.

Our finding suggests that patients receiving certoparin had a comparable low incidence of symptomatic TE, similar to those receiving enoxaparin in combination with IPS, but those treated with enoxaparin without IPS exhibited significantly higher symptomatic TE incidence. This highlights the potential importance of IPS in thromboprophylaxis. While current S3 guideline recommend the use of IPS, no studies have specifically evaluated the combination of IPS with different LMWHs to determine which combination offers the best protection against thromboembolic complications^18,19,21,37^. Further studies are required to investigate whether adding IPS to certoparin or even using IPS alone could lead to similarly effective outcomes. Understanding the interplay between these interventions could refine prophylactic strategies, particularly for high-risk patients. A systematic review by Bianconi et al. (2023)^25^ highlights the complexity of anticoagulation management in patients with high-grade gliomas, where both venous thromboembolism (VTE) and intracranial hemorrhage (ICH) present serious and opposing risks. Their analysis reported VTE rates ranging from 4 to 33% and ICH rates of up to 15.4% in anticoagulated patients, underscoring the delicate balance required in choosing an appropriate thromboprophylaxis strategy. In line with their conclusion, our data emphasize the need for individualized risk stratification and suggest that combining pharmacologic and mechanical prophylaxis may offer a favorable risk–benefit profile. Unlike Bianconi et al., who found limited evidence regarding newer agents and mechanical adjuncts, our findings provide real-world evidence for the additive protective effect of IPS in high-risk neurosurgical patients.

The widely used Khorana score for predicting symptomatic TE in cancer patients has been shown to be inapplicable to patients with GBM^38^. While highly sensitive (98%) for predicting symptomatic TE, its specificity in GBM populations is low (5,6%), as Yust-Katz et al. showed 2014 in their clinical study (*n* = 418)^39^. Khorana himself acknowledged that the data set used to develop the Khorana Score included only a small number of brain tumor patients (*n* = 4). In addition, patients with poor performance status were underrepresented in the cohort, which may limit the applicability of the score to these populations^40^. The Khorana score is made up of the following parameters: (1) cancer type; (2) pre-chemo platelet count; (3) hemoglobin level; (4) pre-chemo leukocyte count; 4) body-mass-index. In 2022, Bell Burdett et al. presented a new predictive time-to-event-model that incorporates additional parameters, to more accurately reflect the complex nature of GBM: (1) history of TE; (2) hypertension; (3) asthma; (4) white blood cell count; (5) WHO tumor grade; (6) patient age; (7) body-mass-index. They created a web based TE prediction tool that was validated in two separate cohorts^41^. Our study confirmed the risk factors hypertension and patients age, but also integrated further risk factors, such as surgery duration and intraoperative blood loss, that could augment Bell’s score by incorporating procedure-specific elements. Future efforts should aim to integrate these factors into a more comprehensive and clinically practical scoring system.

### Clinical implications

The results of this study highlight the need for a personalized approach to thromboprophylaxis in glioblastoma patients undergoing surgery. The significantly lower incidence of symptomatic TE in patients treated with either certoparin or the combination of enoxaparin with IPS, compared to enoxaparin alone, suggests that the choice of anticoagulant and the addition of mechanical prophylaxis can have a meaningful impact on postoperative outcomes. These findings support the routine integration of IPS in high-risk patients and suggest that certoparin may be a more favorable pharmacologic option than enoxaparin in this population.

Moreover, the identification of patient-specific (e.g., diabetes mellitus) and procedure-specific (e.g., prolonged surgery, high intraoperative blood loss) risk factors for TE underlines the importance of individualized prophylactic strategies. In current clinical practice, thromboprophylaxis is often administered uniformly, without taking into account the surgical burden or comorbidities. Our data advocate for a risk-adapted regimen that considers both individual and procedural variables when determining the optimal prophylactic strategy.

In addition, these findings could inform future updates of clinical guidelines, which currently provide only general or conflicting recommendations for thromboprophylaxis in neurosurgical oncology. Implementation of structured risk assessment tools may help clinicians identify patients who benefit most from intensified or combined prophylactic measures, while minimizing the risk of intracranial hemorrhage.

### Limitations and future directions

This study has several limitations. The cohort size was sufficient for identifying statistically significant associations between thromboprophylaxis strategies and TE, as well as for conducting subgroup analyses. Nevertheless, the study has several limitations, including the relatively small absolute number of symptomatic TE events (*n* = 28), potentially limiting the statistical power and generalizability of the results. Additionally, the single-center design and absence of systematic screening for asymptomatic thromboembolic events might have contributed to a detection bias, possibly underestimating the true incidence of TE.

A comparison group for the combination of certoparin and IPS was not feasible due to supply issues with certoparin starting in 2019. Our objective was to compare our in-house prophylaxis measures with each other and to identify risk factors. We were able to achieve meaningful results in this regard. Given the medical context, we focused on clinical parameters.

Nonetheless, future studies should consider incorporating laboratory-based coagulation markers to evaluate the applicability of established predictive models such as the Khorana or Bell score in glioblastoma patients. This would allow for a more refined stratification of thrombotic risk based on objective biomarkers.

Moreover, the development of a glioblastoma-specific, easy-to-use predictive model integrating clinical, procedural, and laboratory parameters could support individualized prophylaxis decisions. In addition, future research should investigate the pharmacokinetic variability among different low-molecular-weight heparins and assess the bioequivalence and clinical efficacy of enoxaparin biosimilars, such as Inhixa and Clexane, which are increasingly used in clinical practice.

## Conclusion

This prospective cohort study demonstrates that thromboprophylaxis strategies differ significantly in their effectiveness among glioblastoma patients undergoing surgical resection. Enoxaparin monotherapy was associated with the highest incidence of postoperative symptomatic thromboembolic events, whereas certoparin and the combination of enoxaparin with intermittent pneumatic stockings (IPS) were linked to lower risk. In addition, clinical and procedural factors such as diabetes mellitus, prolonged surgery duration, and intraoperative blood loss were independently associated with increased thromboembolic risk.

These findings support the need for a more individualized approach to thromboprophylaxis in this vulnerable population. Tailoring preventive strategies to patient- and procedure-specific risk profiles may help reduce thromboembolic complications without increasing the risk of bleeding. Future research should focus on validating glioblastoma-specific risk scores and investigating the comparative efficacy of different LMWHs and their biosimilars in neurosurgical oncology.

## Data Availability

The datasets generated during and/or analyzed during the current study are available from the corresponding author on reasonable request.
